# Differential gene expression in human granulosa cells from recombinant FSH versus human menopausal gonadotropin ovarian stimulation protocols

**DOI:** 10.1186/1477-7827-8-25

**Published:** 2010-03-12

**Authors:** John Brannian, Kathleen Eyster, Breanne A Mueller, Mandi G Bietz, Keith Hansen

**Affiliations:** 1 Department of Obstetrics & Gynecology, Sanford School of Medicine of the University of South Dakota, Sioux Falls SD, USA; 2 Sanford Research/USD Women's Health Research Center, Sioux Falls SD, USA

## Abstract

**Background:**

The study was designed to test the hypothesis that granulosa cell (GC) gene expression response differs between recombinant FSH and human menopausal gonadotropin (hMG) stimulation regimens.

**Methods:**

Females < 35 years-old undergoing IVF for tubal or male factor infertility were prospectively randomized to one of two stimulation protocols, GnRH agonist long protocol plus individualized dosages of (1) recombinant (r)FSH (Gonal-F) or (2) purified human menopausal gonadotropin (hMG; Menopur). Oocytes were retrieved 35 h post-hCG, and GC were collected. Total RNA was extracted from each GC sample, biotinylated cRNA was synthesized, and each sample was run on Human Genome Bioarrays (Applied Microarrays). Unnamed genes and genes with <2-fold difference in expression were excluded.

**Results:**

After exclusions, 1736 genes exhibited differential expression between groups. Over 400 were categorized as signal transduction genes, ~180 as transcriptional regulators, and ~175 as enzymes/metabolic genes. Expression of selected genes was confirmed by RT-PCR. Differentially expressed genes included A kinase anchor protein 11 (AKAP11), bone morphogenetic protein receptor II (BMPR2), epidermal growth factor (EGF), insulin-like growth factor binding protein (IGFBP)-4, IGFBP-5, and hypoxia-inducible factor (HIF)-1 alpha.

**Conclusions:**

Results suggest that major differences exist in the mechanism by which pure FSH alone versus FSH/LH regulate gene expression in preovulatory GC that could impact oocyte maturity and developmental competence.

## Background

Follicle development during the menstrual cycle is directly controlled by gonadotropin (FSH and LH) stimulation from the pituitary, as well as complex paracrine and autocrine regulation within the ovary that is modulated by the hormonal and metabolic environment. FSH and LH elicit receptor-mediated actions directly on granulosa and theca cells of the follicle, which in turn regulate the maturation and development of the oocyte. The oocyte reciprocally communicates with the follicular somatic cells to modulate their activity. In assisted reproductive procedures such as in vitro fertilization (IVF), follicle development is controlled by administration of pharmacologic preparations of human gonadotropins. Human menopausal gonadotropins (hMG) containing both FSH and LH activities, purified from the urine of post-menopausal women, have been used successfully in the clinical setting for many years. Advances in purification techniques have led to a new generation of hMG preparations that are very consistent in their FSH and LH bio-activities [1]. In recent years, genetic engineering technology has allowed for the production of recombinant (r)FSH. These preparations are extremely pure and have no LH activity [2].

Both hMG and rFSH stimulation protocols are widely used in clinical ART programs. Although neither of these strategies perfectly mimics the natural ovarian environment, both yield good pregnancy outcomes. Nevertheless physiologists and clinicians continue to debate which stimulation type yields oocytes with optimal developmental competence. Studies comparing the efficacy of rFSH versus hMG stimulation have generally focused on pregnancy and implantation rates [3-7]. Results of these studies, even those of prospective, randomized design, are inconsistent and difficult to interpret because of the many confounding variables that affect outcomes. Very few studies have addressed the impact of specific gonadotropin preparations on the cellular physiology of follicular cells.

Even though FSH and LH both act primarily via cAMP/PKA-mediated pathways, they are clearly not identical in their signaling pathways and gene targets [8]. Moreover, both FSH and LH interact with other ligand-receptor pathways, e.g. IGF, that modulate their actions, and are dependent upon the specific stage of the cycle. For example, FSH activates protein kinase B/Akt and glucocorticoid-induced kinase in growing follicles, whereas LH induces progesterone receptor in preovulatory granulosa cells [8].

DNA microarray technology can screen the relative expression of the human genome from a single mRNA extract of tissue or isolated cells. The purpose of this study was to test the hypothesis that granulosa cell (GC) gene expression response differs between pure recombinant FSH and human menopausal gonadotropin (hMG) stimulation regimens. If so, differences in gene expression may subsequently reveal altered signaling, transcription/translation, or metabolic pathways that could impact oocyte maturation and developmental competence.

## Methods

### Patient selection & tissue acquisition

The study was approved by the University of South Dakota Institutional Review Board, and all participants signed informed consent. Female patients (n = 8; 4 per treatment) under 35 years-old undergoing IVF for tubal or male factor infertility were prospectively randomized to one of two stimulation protocols, GnRH agonist (Lupron^®^, TAP Pharmaceutical) long protocol plus (1) recombinant (r)FSH (Gonal-F^®^, Serono) or (2) purified human menopausal gonadotropin (hMG; Menopur^®^; Ferring, 75 IU FSH/75 IU LH activity per vial). Patients received individualized dosages of only rFSH or only hMG. Follicle development was monitored by ultrasonography and serum estradiol concentrations. When at least two follicles were ≥ 17 mm, hCG (10,000 IU; Novarel^®^, Ferring) was administered.

Oocytes were retrieved 35 hours post-hCG. All follicles ≥15 mm in diameter were aspirated into HEPES-buffered HTF (SAGE^®^). Immediately following oocyte recovery from each aspirate, remaining fluid was collected in 50-ml conical tubes containing RNALater^® ^(Applied Biosystems) and kept on ice until the end of the retrieval. Pooled aspirates from all follicles were centrifuged, washed once, and re-suspended in approximately 2 ml HEPES-buffered HTF, and then centrifuged over 40% Percoll^® ^(Sigma) gradients to remove the majority of red blood cells (RBC). Each overlay was washed in 4-5 volumes of HEPES-buffered HTF. The final pellet was re-suspended in 1 ml RNALater^®^, and snap-frozen in liquid nitrogen.

### RNA extraction

Total RNA was extracted from each individual patient sample. For extraction of RNA, GC were submerged in TRI reagent (Molecular Research Center) and homogenized. After homogenization, 105 μl 3 M sodium acetate, and 350 μl bromochloropropane was added to the supernate and mixed, and the sample was then incubated on ice for 15 min. The sample was centrifuged and the aqueous layer was removed and purified on an RNeasy column (Qiagen). The sample was treated with an on-column RNase-free DNase (Qiagen) to remove any contaminating genomic DNA. The total RNA sample was then eluted from the column. The RNA quantity and purity of the sample was analyzed using the RNA 6000 Nano Lab chip on an Agilent Bioanalyzer (Agilent Technologies). Total RNA obtained in individual samples ranged from 0.3 μg to 47.6 μg. The RNA extracts were stored at -70°C until all samples were collected.

### DNA microarrays

Total RNA extracts were labeled and run individually on CodeLink Whole Human Genome Bioarrays (GE-Amersham Biosciences). These microarrays contain 53,000 single-stranded 30-mer oligonucleotide probes for human genes. For the labeling reaction, first-strand cDNA was reverse transcribed from the total RNA sample and second-strand cDNA was synthesized from the first cDNA strand. Complementary RNA was synthesized from the cDNA; this reaction incorporated biotin-11-UTP into the cRNA. The synthesis of first and second strand cDNA and synthesis of cRNA used reagents from the CodeLink Expression Assay Reagent Kit (GE-Amersham Biosciences) according to the manufacturer's instructions. One sample (hMG group) yielded poor RNA recovery and was excluded from microarray analysis.

The biotinylated cRNA was purified on Qiagen RNeasy columns, fragmented, and hybridized with the DNA microarrays for 18 hours at 37°C. The hybridized slides were washed, incubated with streptavidin-Alexa Fluor 647 (Molecular Probes/Invitrogen) to label the biotinylated cRNA hybridized to the slides, and washed again. The slides were scanned with an Axon GenePix Scanner and analyzed with GenePix Pro (MDS Analytical Technologies), CodeLink (GE-Amersham), Acuity (MDS Technologies) and GeneSpring (Agilent Technologies) software. The GenePix Pro software was used to obtain the microarray image. CodeLink software applied the background correction. GeneSpring software was used to normalize the expression of each gene to the median gene expression and each slide to the 50^th ^percentile of gene expression and to perform statistical analysis. The data set for these DNA microarrays has been deposited at the National Center for Biotechnology Information Gene Expression Omnibus [GEO; [9]] as recommended by Minimum Information about a Microarray Experiment [MIAME] standards (accession number GSE16523).

### Real time RT-PCR

Pre-designed primers and fluorescent (FAM) labeled minor groove binding probes were obtained from Applied Biosystems. Real time RT-PCR was carried out with TaqMan Gold RT-PCR reagents (Applied Biosystems) as described [10]. Changes in relative expression of genes of interest were calculated; data were normalized to an endogenous control (GAPDH). An RNA concentration-response validation curve was carried out to determine the concentration of RNA to add to the RT-PCR reaction. All samples were run in duplicate. The Relative Expression Software Tool (REST^©^) [11] was used for statistical analysis of the data from the real time RT-PCR reaction. This analytical tool incorporates the variability of data from both the housekeeping gene, GAPDH, as well as that of the genes of interest when calculating statistical significance.

### Statistical analysis

GeneSpring software (Agilent Technologies) was used to compare relative gene expression. This program performed a t-test on the data for statistical analysis of the DNA microarrays; p value was set at 0.05. Those genes shown to be differentially expressed were sorted by function; each gene was placed into only one gene ontology. Unnamed genes were not further analyzed. Genes for which the average expression values for both rFSH and hMG were both less than 0.2 were also excluded from further analysis, as were genes with < 2-fold difference in expression between groups.

## Results and Discussion

There were no differences in patient age, BMI, basal FSH level, days of stimulation, number of mature follicles, number of oocytes retrieved, or clinical pregnancy rate between treatment groups (Table 1).

**Table 1:** Patient demographics (n = 4 rFSH; n = 3 hMG; mean ± SEM). (*NS *= not statistically significant.)

	rFSH	hMG	significance
Age	29.8 ± 2.1	28.7 ± 4.1	NS
BMI	22.2 ± 0.9	22.3 ± 0.3	NS
Basal FSH	6.3 ± 0.4	6.7 ± 0.8	NS
Days of stimulation	9.3 ± 0.5	9.5 ± 1.1	NS
No. follicles ≥15 mm	12.3 ± 3.1	11.0 ± 4.0	NS
No. oocytes retrieved	13.5 ± 3.2	10.3 ± 3.3	NS
Total IU FSH	3337 ± 164	2900 ± 704	NS
Clinical Pregnancy Rate	2/4	2/3	NS

**Figure 1:**
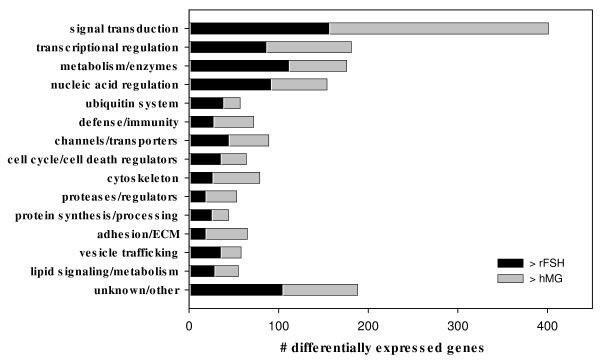
**Differentially expressed genes by functional category**. Black bars represent genes with greater expression in rFSH-stimulated GC (n = 4); gray bars represent genes with greater expression in hMG-stimulated GC (n = 3).

**Figure 2:**
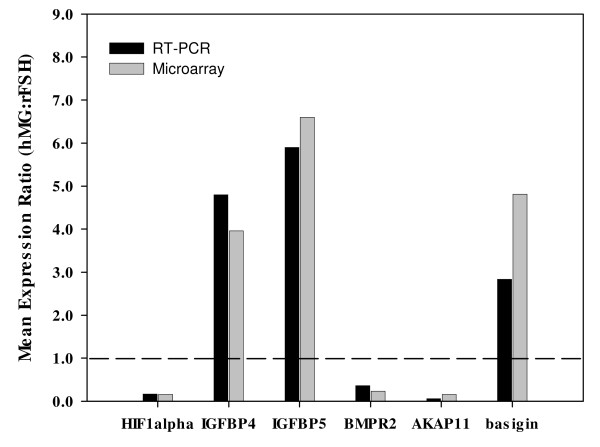
**Comparison of mean relative (hMG:rFSH) gene expression of selected genes by DNA microarray (gray bars) and RT-PCR (black bars)**. The expression of each gene of interest was calculated relative to that of GAPHD, then the expression in hMG-treated samples was divided by that of the rFSH-treated samples. For both sets of data, the values represent the ratios of the group means.

After the exclusions noted above, 1736 genes exhibited ≥ 2-fold differential expression between groups. Over 400 of these were categorized as signal transduction genes, ~180 as transcriptional regulators, and ~175 as enzymes/metabolic genes (Figure 1). A condensed list of differentially expressed genes by functional category is shown in Additional File 1, Table S1. The complete list of differentially expressed genes is shown in Additional File 2, Table S2. The entire data set can be accessed at GEO [9] using accession number GSE16523. Expression of selected genes was confirmed by RT-PCR. Relative expression by microarray and PCR were generally similar (Figure 2).

The results demonstrate that gene expression in GC of periovulatory follicles is highly dependent upon specific gonadotropin regimens used for controlled ovarian stimulation, and confirm a recent report by Grǿndahl, *et al. *[12]. The present data are surprising in the large number and functional diversity of genes that differ in expression between recombinant FSH and hMG protocols. The major difference between rFSH and hMG preparations is the absence or presence of LH activity. However, there are also differences in the FSH isoform profiles and potentially in specific FSH activity that may contribute to the differences in gene expression observed. Discerning Differences in GC gene expression have broad implications for oocyte maturation, early embryo development, and clinical outcomes in ART patients. The present results open the door for a new line of investigation into the impact of GC gene regulation on oocyte function.

GC were collected for analysis 35 h after administration of a standard dose of hCG, and were therefore undergoing luteinization. The profound differences in gene expression are all the more striking considering that hCG was the dominant hormonal influence during the preceding 35 hours in both groups. It is possible that the hMG group had accelerated luteinization relative to the rFSH group due to prior LH exposure. For example, 3-beta-hydroxysteroid dehydrogenase, *IGFBP-4*, *IGFBP-5*, basigin, *HIF-1alpha*, vascular endothelial growth factor (*VEGF*), and related genes were expressed at greater levels in the hMG group, changes expected during luteinization [13-18]. This suggests that many, if not all, of the observed differences in gene expression in GC were a manifestation of a distinct response to the ovulatory/luteinizing stimulus that was determined by the type of follicular stimulation the cells were exposed to. It would be very interesting to repeat this experiment in a non-human primate model where GC could be collected without hCG administration [19,20].

The largest groups of differentially expressed genes were those related to signal transduction and transcriptional regulation. *AKAP11*, for example, was much more highly expressed in rFSH-stimulated cells than in hMG-stimulated cells. AKAP11 is a protein kinase A (PKA) anchor protein, which binds both RI and RII PKA regulatory subunits, targeting PKA to specific intracellular sites [21]. Although not studied in the ovary, AKAP11 is highly expressed in the human testis where it is thought to play an important role in spermatogenesis [22].

In addition to numerous signaling proteins involved in cAMP signaling pathways, genes involved in other signaling pathways were differentially expressed. For example, signal transducer and activator of transcription (*STAT*) 5B and *STAT 6*, suppressor of cytokine signaling (*SOCS*) 1 and *SOCS 5*, and several members of mitogen-activated protein (MAP) kinase cascades. Numerous receptor genes were differentially expressed, e.g. bone morphogenetic protein (*BMP*) receptors type 1A, 1B, and 2, adiponectin receptor, and histamine (*H1*) receptor. There were also genes encoding signaling ligands that differed between groups, such as growth differentiation factor (*GDF*) 15, *GDF 11*, transforming growth factor B1 (*TGFB1*), and epidermal growth factor (*EGF*). The results demonstrate that differences in gonadotropin stimulation can significantly impact multiple aspects of regulatory granulosa cell signaling pathways. It remains to be elucidated how these differences may affect oocyte and luteal function.

Among the differentially expressed genes were several with known importance to oocyte maturation. Notably, the BMP family receptors (*BMPR1A, BMPR1B, BMPR2*), are the receptors for GDF-9 and BMP-15 [23,24]. GDF-9 and BMP-15 are oocyte-derived factors that are major mediators of oocyte-granulosa cell communication during folliculogenesis and the periovulatory period [25,26]. The five-fold reduction in *BMPR2 *expression in hMG-stimulated GC relative to rFSH-stimulated GC suggests that they may be less responsive to GDF-9, at least at the time point sampled. Consistent with lesser *BMPR2 *expression, the gene encoding the downstream signaling protein SMAD5 was 3-fold less in hMG-stimulated GC. Whether these differences indicate a fundamental difference in function, or represent only a temporal difference in the normal sequence of periovulatory events is not known. Nevertheless, even a temporal difference in GDF-9/BMP-15 signaling could have significant effects on oocyte quality and developmental potential.

Moreover, the differential expression of certain key metabolic enzyme genes implies a major shift in metabolic pathways. For example, hMG-stimulated GC had a more than a 5-fold greater expression of pyruvate kinase, whereas rFSH-stimulated GC had greater expression of malate dehydrogenase and lactate dehydrogenase. The oocyte derives most of its cellular energy from pyruvate synthesized via glycolysis in cumulus GC, which is regulated by oocyte-derived factors such as BMP-15, GDF-9, and fibroblast growth factor (FGF) [27]. Differences in BMP-15/GDF-9 signaling may be directly related to differential metabolic gene expression in hMG- versus rFSH-stimulated GC. Although the majority of GC collected in the present study were mural GC, the results suggest possible differences in oocyte energy substrate availability, which could impact oocyte developmental competence.

In a recent similar study [12], only 85 genes were found to be differentially expressed in GC from women stimulated with rFSH or hMG. Pooling of patient samples, use of a different microarray system, and use of different statistical software [12] may account for differences in results. However, among the genes identified in that study were several associated with lipid metabolism and protein signaling/phosphorylation, as in the present study. Differentially expressed genes that were commonly identified in both studies include 3-hydroxy-3-methyl-Coenzyme A synthase 1, fibrinogen gamma polypeptide, phosphoinositide-3-kinase alpha polypeptide, *SOCS 1*, transferrin receptor (CD71), and multiple protein tyrosine phosphatases (non-receptor type).

Future studies will be directed towards more in depth investigation of the regulation and function of some of the genes and gene pathways revealed in this study. For example, cell culture studies performed under more controlled in vitro conditions may distinguish between temporal differences in gene expression related to GC maturation in response to FSH and LH versus more fundamental differences in FSH and LH signaling. Moreover, genes not previously studied in the ovary may reveal important new regulatory mechanisms.

## Conclusions

GC recovered from IVF patients at oocyte retrieval who were stimulated with either rFSH or hMG displayed strikingly different profiles of gene expression. Some or all of these differences may reflect divergent timing of periovulatory events. Nevertheless, considering our current understanding of the intimate relationship between GC and oocyte function, differential gene expression in GC strongly implies that gonadotropin stimulation protocols for IVF could have a major impact on oocyte functional status. The present results should open up new lines of discussion and research regarding optimal gonadotropin stimulation for ART, as well as for better understanding fundamental aspects of folliculogenesis, ovulation, oocyte maturation, and luteinization.

## Competing interests

The authors declare that they have no competing interests.

## Authors' contributions

JB, KE, and KH conceived and designed the study. KH conducted the clinical treatment. BAM and MGB performed the RNA extractions, assisted with the microarrays, and prepared preliminary data summaries. BAM wrote the preliminary draft of the manuscript. KE supervised RNA extractions and microarray analyses, and performed statistical analyses on microarray data. JB performed final data analysis and wrote the final manuscript. All authors read and approved the final manuscript.

## Supplementary Material

Additional file 1 Condensed list of differentially expressed genes. Click here for file

Additional file 2 Complete list of differentially expressed genes. Click here for file
