# Supplementary material for: Differential gene expression in human granulosa cells from recombinant FSH versus human menopausal gonadotropin ovarian stimulation protocols
Source: Reprod Biol Endocrinol. 2010 Mar 12;8:25. doi: 10.1186/1477-7827-8-25 (PMC2842272; doi:10.1186/1477-7827-8-25)
Supplement: Additional file 1 — Condensed list of differentially expressed genes. [file 1477-7827-8-25-S1.PDF]

Table S1. Condensed list of differentially expressed genes.

hMG:rFSH (mean ratio)	rFSH (normalized expression)	hMG	p =	accession #	gene name
**signal transduction**					
0.16	48.38	7.78	0.02	NM_016248.2	A kinase (PRKA) anchor protein 11 (AKAP11)
0.21	29.47	6.07	0.04	NM_002748.2	mitogen-activated protein kinase 6 (MAPK6)
0.21	10.48	2.22	0.02	NM_144949.2	suppressor of cytokine signaling 5 (SOCS5)
0.23	21.79	5.02	0.03	NM_015387.2	preimplantation protein 3 (PREI3)
0.23	8.03	1.85	0.00	NM_033346.2	bone morphogenetic protein receptor, type II (BMPR2)
0.24	11.33	2.71	0.00	NM_024745.2	SHC SH2-domain binding protein 1 (SHCBP1)
0.26	5.90	1.54	0.01	NM_002830.2	protein tyrosine phosphatase, non-receptor type 4 (PTPN4)
0.30	2.68	0.80	0.01	NM_001203.1	bone morphogenetic protein receptor, type IB (BMPR1B)
0.31	5.89	1.84	0.00	NM_004329.2	bone morphogenetic protein receptor, type IA (BMPR1A)
0.32	28.81	9.16	0.01	NM_002710.1	protein phosphatase 1, catalytic subunit, gamma isoform (PPP1CC)
0.34	5.06	1.70	0.03	NM_000560.2	CD53 antigen (CD53)
0.34	4.66	1.58	0.02	NM_001981.1	epidermal growth factor receptor pathway substrate 15 (EPS15)
0.35	123.01	42.60	0.03	NM_005625.1	syndecan binding protein (syntenin) (SDCBP)
0.35	10.71	3.74	0.04	NM_001001419.1	SMAD, mothers against DPP homolog 5 (SMAD5)
0.35	254.20	90.08	0.02	NM_015161.1	ADP-ribosylation factor-like 6 interacting protein (ARL6IP)
0.36	4.82	1.75	0.00	NM_002716.3	protein phosphatase 2, regulatory subunit A (PR 65), beta isoform (PPP2R1B)
0.38	11.13	4.21	0.01	NM_014110.3	protein phosphatase 1, regulatory (inhibitor) subunit 8 (PPP1R8)
0.39	34.39	13.55	0.04	NM_000210.1	integrin, alpha 6 (ITGA6)
0.40	30.09	11.89	0.03	NM_005109.1	oxidative-stress responsive 1 (OSR1)
0.40	9.22	3.72	0.03	NM_024551.2	adiponectin receptor 2 (ADIPOR2)
0.41	6.82	2.83	0.02	NM_003264.2	toll-like receptor 2 (TLR2)
0.45	17.73	7.99	0.00	NM_005506.2	scavenger receptor class B, member 2 (SCARB2)
1.98	2.41	4.78	0.05	NM_012448.3	signal transducer and activator of transcription 5B (STAT5B)
2.24	11.54	25.80	0.05	NM_005501.1	integrin, alpha 3 (antigen CD49C, alpha 3 subunit of VLA-3 receptor) (ITGA3)
2.24	6.01	13.47	0.02	NM_000861.2	histamine receptor H1 (HRH1)
2.60	4.44	11.53	0.02	NM_006129.2	bone morphogenetic protein 1 (BMP1), transcript variant BMP1-3
2.63	2.09	5.50	0.00	NM_199054.1	MAP kinase-interacting serine/threonine kinase 2 (MKNK2)
2.68	11.46	30.72	0.01	NM_183001.2	SHC (Src homology 2 domain containing) transforming protein 1 (SHC1)
2.75	14.86	40.84	0.00	NM_001894.4	casein kinase 1, epsilon (CSNK1E)
2.79	3.24	9.04	0.04	NM_006301.2	mitogen-activated protein kinase kinase kinase 12 (MAP3K12)
2.83	3.72	10.52	0.01	NM_002229.2	jun B proto-oncogene (JUNB)
2.83	2.51	7.11	0.00	NM_001784.2	CD97 antigen (CD97)
2.86	26.99	77.27	0.03	NM_016639.1	tumor necrosis factor receptor superfamily, member 12A (TNFRSF12A)
2.96	6.91	20.44	0.04	NM_004864.1	growth differentiation factor 15 (GDF15)
2.99	1.27	3.80	0.00	NM_000603.3	nitric oxide synthase 3 (endothelial cell) (NOS3)
3.26	0.92	3.02	0.01	NM_003745.1	suppressor of cytokine signaling 1 (SOCS1)
3.31	11.24	37.23	0.04	NM_031286.2	SH3 domain binding glutamic acid-rich protein like 3 (SH3BGRL3)
3.43	7.04	24.15	0.03	NM_005866.2	opioid receptor, sigma 1 (OPRS1)
3.45	8.24	28.44	0.05	NM_001963.2	epidermal growth factor (beta-urogastrone) (EGF)
3.45	19.71	68.01	0.01	NM_004494.1	hepatoma-derived growth factor (high-mobility group protein 1-like) (HDGF)
3.46	0.41	1.41	0.01	NM_005811.2	growth differentiation factor 11 (GDF11)
3.71	3.16	11.71	0.00	NM_145110.1	mitogen-activated protein kinase kinase 3 (MAP2K3)
3.86	1.56	6.04	0.00	NM_001953.2	endothelial cell growth factor 1 (platelet-derived) (ECGF1)
3.90	5.18	20.18	0.02	NM_016547.1	calcium binding protein Cab45 precursor (Cab45)
3.93	0.48	1.87	0.04	NM_000020.1	activin A receptor type II-like 1 (ACVRL1)
3.93	5.79	22.75	0.00	NM_001552.1	insulin-like growth factor binding protein 4 (IGFBP4)
3.96	2.61	10.35	0.00	NM_002840.2	protein tyrosine phosphatase, receptor type, F (PTPRF)
4.10	1.62	6.63	0.04	NM_003153.3	signal transducer and activator of transcription 6, interleukin-4 induced (STAT6)
4.13	2.07	8.54	0.02	NM_004295.2	TNF receptor-associated factor 4 (TRAF4)
4.51	1.33	5.97	0.00	NM_000476.1	adenylate kinase 1 (AK1)
4.81	7.03	33.82	0.03	NM_001728.2	basigin (OK blood group) (BSG)
5.02	3.87	19.45	0.01	NM_005860.1	follistatin-like 3 (secreted glycoprotein) (FSTL3)
5.21	1.77	9.25	0.02	NM_001157.2	annexin A11 (ANXA11)
5.38	2.45	13.18	0.00	NM_006293.2	TYRO3 protein tyrosine kinase (TYRO3)
5.48	1.79	9.83	0.00	NM_002205.1	integrin, alpha 5 (fibronectin receptor, alpha polypeptide) (ITGA5)

5.61	7.87	44.19	0.00	NM_033256.1	protein phosphatase 1, regulatory (inhibitor) subunit 14A (PPP1R14A)
5.79	2.68	15.53	0.04	NM_033133.3	2',3'-cyclic nucleotide 3' phosphodiesterase (CNP)
6.17	1.47	9.05	0.01	NM_000660.1	transforming growth factor, beta 1 (TGFB1)
6.19	1.69	10.45	0.04	NM_002419.2	mitogen-activated protein kinase kinase kinase 11 (MAP3K11)
6.28	1.48	9.27	0.00	NM_007346.2	opioid growth factor receptor (OGFR)
6.30	3.02	19.00	0.04	NM_003377.3	vascular endothelial growth factor B (VEGFB)
6.40	3.84	24.60	0.01	NM_004356.2	CD81 antigen (target of antiproliferative antibody 1) (CD81)
6.64	4.61	30.59	0.02	NM_013403.1	striatin, calmodulin binding protein 4 (STRN4)
6.66	3.32	22.11	0.00	NM_000599.2	insulin-like growth factor binding protein 5 (IGFBP5)
6.74	1.61	10.84	0.02	NM_021923.2	fibroblast growth factor receptor-like 1 (FGFRL1)
6.83	8.20	55.96	0.04	NM_004712.3	hepatocyte growth factor-regulated tyrosine kinase substrate (HGS)
8.44	5.33	45.01	0.04	NM_004357.3	CD151 antigen (CD151)
8.58	2.26	19.38	0.03	NM_006453.2	transducin (beta)-like 3 (TBL3)
11.49	2.25	25.87	0.04	NM_000120.2	epoxide hydrolase 1, microsomal (xenobiotic) (EPHX1)
12.45	2.23	27.79	0.02	NM_003258.1	thymidine kinase 1, soluble (TK1)
14.84	2.62	38.87	0.04	NM_182486.1	C1q and tumor necrosis factor related protein 6 (C1QTNF6)
19.17	0.25	4.86	0.02	NM_139314.1	angiopoietin-like 4 (ANGPTL4)
**lipid signaling/metabolism**					
0.15	21.21	3.16	0.05	NM_024420.1	phospholipase A2, group IVA (cytosolic, calcium-dependent) (PLA2G4A)
0.17	15.69	2.60	0.00	NM_181523.1	phosphoinositide-3-kinase, regulatory subunit, polypeptide 1 (p85 alpha) (PIK3R1)
0.20	14.73	2.95	0.05	NM_005536.2	inositol(myo)-1(or 4)-monophosphatase 1 (IMPA1)
0.33	17.88	5.90	0.03	NM_003129.2	squalene epoxidase (SQLE)
0.33	34.25	11.41	0.01	NM_006745.2	sterol-C4-methyl oxidase-like (SC4MOL)
3.71	17.43	64.70	0.03	NM_182676.1	phospholipid transfer protein (PLTP)
4.62	5.56	25.67	0.04	NM_025194.2	inositol 1,4,5-trisphosphate 3-kinase C (ITPKC)
5.93	4.85	28.75	0.02	NM_014216.3	inositol 1,3,4-triphosphate 5/6 kinase (ITPK1)
6.27	0.85	5.31	0.02	NM_005027.2	phosphoinositide-3-kinase, regulatory subunit, polypeptide 2 (p85 beta) (PIK3R2)
12.01	6.65	79.86	0.04	NM_012268.1	phospholipase D3 (PLD3)
26.67	6.18	164.80	0.02	NM_000041.2	apolipoprotein E (APOE)
**transcriptional regulation**					
0.16	48.95	7.63	0.05	NM_006746.3	sex comb on midleg-like 1 (SCML1) X chromosome
0.21	40.33	8.42	0.03	BC037828.1	general transcription factor IIA, 1, 19/37kDa
0.22	10.74	2.31	0.03	NM_014733.2	zinc finger, FYVE domain containing 16 (ZFYVE16)
0.24	45.60	10.72	0.01	NM_015555.1	zinc finger protein 451 (ZNF451)
0.30	82.43	24.41	0.02	NM_001530.2	hypoxia-inducible factor 1, alpha subunit (HIF1A)
0.30	33.82	10.26	0.04	NM_017569.2	transcription factor (p38 interacting protein) (P38IP)
0.33	11.15	3.64	0.02	NM_016107.3	zinc finger RNA binding protein (ZFR)
0.35	16.28	5.62	0.00	NM_183011.1	cAMP responsive element modulator (CREM)
2.33	5.03	11.75	0.01	NM_004474.1	forkhead box D2 (FOXD2)
3.04	2.73	8.30	0.00	NM_016545.3	immediate early response 5
4.00	8.34	33.39	0.02	NM_005253.3	FOS-like antigen 2 (FOSL2)
4.16	5.22	21.71	0.00	NM_016202.2	zinc finger protein 580 (ZNF580)
6.84	1.74	11.90	0.03	NM_022749.4	retinoic acid induced 16 (RAI16)
7.71	1.56	12.05	0.00	NM_004078.1	cysteine and glycine-rich protein 1 (CSRP1)
9.55	1.80	17.19	0.04	NM_020680.2	SCY1-like 1 (SCYL1) [telomerase regulation-associated protein]
**metabolism/enzymes**					
0.09	8.06	0.72	0.00	AI088935.1	similar to STEROL O-ACYLTRANSFERASE
0.13	8.65	1.16	0.01	NM_004665.2	vanin 2 (VNN2)
0.17	22.57	3.75	0.01	NM_002130.3	3-hydroxy-3-methylglutaryl-Coenzyme A synthase 1 (soluble) (HMGCS1)
0.18	72.34	13.13	0.01	NM_000153.1	galactosylceramidase (Krabbe disease) (GALC)
0.20	73.96	14.51	0.03	NM_001634.3	adenosylmethionine decarboxylase 1 (AMD1)
0.20	42.24	8.49	0.00	NM_001086.1	arylacetamide deacetylase (esterase) (AADAC)
0.21	29.70	6.30	0.02	NM_000859.1	3-hydroxy-3-methylglutaryl-Coenzyme A reductase (HMGCR)
0.26	26.78	6.84	0.01	NM_016371.1	hydroxysteroid (17-beta) dehydrogenase 7 (HSD17B7)
0.29	303.37	87.37	0.00	NM_005917.2	malate dehydrogenase 1, NAD (soluble) (MDH1)
0.32	67.49	21.30	0.01	NM_000849.3	glutathione S-transferase M3 (brain) (GSTM3)
0.33	68.20	22.79	0.00	NM_000235.1	lipase A, lysosomal acid, cholesterol esterase (Wolman disease) (LIPA)
0.35	69.35	24.37	0.01	NM_005891.1	acetyl-Coenzyme A acetyltransferase 2 (acetoacetyl Coenzyme A thiolase) (ACAT2)
0.38	21.17	8.00	0.00	NM_000786.2	cytochrome P450, family 51, subfamily A, polypeptide 1 (CYP51A1)
0.40	310.01	125.32	0.00	NM_002300.3	lactate dehydrogenase B (LDHB)
0.45	63.94	28.59	0.02	NM_007099.2	acid phosphatase 1, soluble (ACP1)

0.47	3.87	1.80	0.04	NM_000414.1	hydroxysteroid (17-beta) dehydrogenase 4 (HSD17B4)
0.50	104.01	51.49	0.03	NM_021074.1	NADH dehydrogenase (ubiquinone) flavoprotein 2, 24kDa (NDUFV2)
0.50	31.36	15.75	0.03	NM_000237.1	lipoprotein lipase (LPL)
3.55	4.07	14.46	0.01	NM_152359.1	carnitine palmitoyltransferase 1C (CPT1C)
4.45	4.46	19.81	0.02	NM_005700.2	dipeptidylpeptidase 3 (DPP3)
5.53	52.69	291.39	0.03	NM_000398.3	diaphorase (NADH) (cytochrome b-5 reductase) (DIA1)
5.77	8.72	50.31	0.04	NM_182470.1	pyruvate kinase, muscle (PKM2)
5.89	0.49	2.86	0.05	NM_025193.2	hydroxy-delta-5-steroid dehydrogenase, 3 beta- and steroid delta-isomerase 7 (HSD3B7)
6.28	2.08	13.07	0.04	NM_005817.2	mannose-6-phosphate receptor binding protein 1 (M6PRBP1)
7.36	4.91	36.10	0.02	NM_015937.2	phosphatidylinositol glycan, class T (PIGT)
7.55	2.66	20.08	0.01	NM_000199.2	N-sulfoglucosamine sulfohydrolase (sulfamidase) (SGSH)
**adhesion/ECM**					
0.13	50.04	6.59	0.00	NM_000509.3	fibrinogen, gamma polypeptide (FGG)
0.20	6.38	1.24	0.01	NM_024422.2	desmocollin 2 (DSC2)
0.32	34.14	10.87	0.04	NM_201515.1	periphilin 1 (PPHLN1)
2.15	6.93	14.90	0.00	NM_000442.2	platelet/endothelial cell adhesion molecule (CD31 antigen) (PECAM1)
2.24	5.67	12.70	0.02	NM_016174.3	cerebral endothelial cell adhesion molecule 1 (CEECAM1)
3.86	3.26	12.56	0.00	NM_022356.2	leucine proline-enriched proteoglycan (leprecan) 1 (LEPRE1)
4.26	2.16	9.22	0.00	NM_002230.1	junction plakoglobin (JUP)
6.28	6.37	40.05	0.04	NM_002292.2	laminin, beta 2 (laminin S) (LAMB2)
7.22	0.49	3.52	0.04	NM_016580.2	protocadherin 12 (PCDH12)
8.01	3.92	31.41	0.02	NM_000302.2	procollagen-lysine, 2-oxoglutarate 5-dioxygenase (PLOD)
8.08	6.78	54.77	0.00	NM_001846.1	collagen, type IV, alpha 2 (COL4A2)
8.76	1.53	13.41	0.02	NM_012445.1	spondin 2, extracellular matrix protein (SPON2)
10.99	0.61	6.65	0.03	NM_005560.3	laminin, alpha 5 (LAMA5)
18.50	0.49	9.02	0.04	NM_005529.2	heparan sulfate proteoglycan 2 (perlecan) (HSPG2)
**nucleic acid regulation**					
0.19	63.16	11.75	0.01	NM_015450.1	protection of telomeres 1 (POT1)
0.23	82.61	18.90	0.03	NM_001356.2	DEAD (Asp-Glu-Ala-Asp) box polypeptide 3, X-linked (DDX3X)
0.24	27.06	6.37	0.02	NM_006756.2	transcription elongation factor A 1 isoform 1
0.42	36.61	15.19	0.00	NM_152858.1	Wilms tumor 1 associated protein (WTAP), transcript variant 3
3.33	1.39	4.64	0.02	NM_004618.2	topoisomerase (DNA) III alpha (TOP3A)
4.38	2.39	10.49	0.05	NM_019100.3	DNA methyltransferase 1 associated protein 1 (DMAP1)
4.79	11.16	53.44	0.00	NM_003089.3	small nuclear ribonucleoprotein 70kDa polypeptide (RNP antigen) (SNRP70)
9.53	2.46	23.42	0.05	NM_005035.2	polymerase (RNA) mitochondrial (DNA directed) (POLRMT)
**protein synthesis/processing**					
0.17	30.05	5.04	0.02	NM_012424.2	ribosomal protein S6 kinase, 52kDa, polypeptide 1 (RPS6KC1)
0.20	8.92	1.77	0.00	NM_004755.2	ribosomal protein S6 kinase, 90kDa, polypeptide 5 (RPS6KA5)
0.20	54.47	11.01	0.00	NM_015317.1	pumilio homolog 2 (PUM2)
0.26	29.30	7.70	0.02	NM_004730.1	eukaryotic translation termination factor 1 (ETF1)
0.29	95.41	27.72	0.04	NM_001349.2	aspartyl-tRNA synthetase
0.30	50.49	14.94	0.01	NM_001967.2	eukaryotic translation initiation factor 4A, isoform 2 (EIF4A2)
4.35	35.74	155.36	0.00	NM_001961.2	eukaryotic translation elongation factor 2 (EEF2)
6.19	5.80	35.86	0.03	NM_004095.2	eukaryotic translation initiation factor 4E binding protein 1 (EIF4EBP1)
7.43	3.98	29.54	0.00	NM_006295.1	valyl-tRNA synthetase 2 (VARS2)
8.71	4.09	35.66	0.01	NM_006184.3	nucleobindin 1 (NUCB1)
**proteases/regulators**					
0.13	4.55	0.59	0.01	NM_006144.2	granzyme A (granzyme 1, cytotoxic T-lymphocyte-associated serine esterase 3) (GZMA)
0.27	79.11	21.54	0.03	NM_002788.2	proteasome (prosome, macropain) subunit, alpha type, 3 (PSMA3)
0.28	47.25	13.38	0.01	NM_016302.2	cereblon (CRBN)
0.39	57.60	22.50	0.00	NM_005857.2	zinc metalloproteinase (STE24 homolog) (ZMPSTE24)
2.15	8.72	18.72	0.05	NM_004390.2	cathepsin H (CTSH)
3.18	4.26	13.54	0.03	NM_004995.2	**matrix metalloproteinase 14 (membrane-inserted) (MMP14) see basigin**
3.94	6.17	24.30	0.03	NM_001749.1	calpain, small subunit 1 (CAPNS1)
6.72	19.28	129.51	0.03	NM_053044.2	serine protease HTRA3 (HTRA3)
8.12	2.24	18.18	0.00	NM_003255.2	**tissue inhibitor of metalloproteinase 2 (TIMP2)**
10.80	5.40	58.28	0.00	NM_001235.2	serine (or cysteine) proteinase inhibitor, clade H (heat shock protein 47) (SERPINH1)
**cytoskeleton**					

0.22	8.54	1.87	0.00	NM_002670.1	plastin 1 (I isoform) (PLS1)
0.22	8.47	1.90	0.05	NM_006751.3	sperm specific antigen 2 (SSFA2)
0.24	16.81	3.98	0.05	NM_016008.2	dynein 2 light intermediate chain (D2LIC)
0.24	14.62	3.56	0.00	NM_016221.2	dynactin 4 (p62) (DCTN4)
4.15	1.67	6.94	0.01	NM_030983.1	microtubule-associated protein 4 (MAP4)
5.74	2.43	13.96	0.00	NM_023019.1	dynactin 1 (p150, glued homolog) (DCTN1)
7.75	5.82	45.09	0.00	NM_005572.2	lamin A/C (LMNA)
15.80	2.68	42.38	0.00	NM_181526.1	myosin, light polypeptide 9, regulatory (MYL9)
18.99	1.28	24.38	0.00	NM_001456.1	filamin A, alpha (actin binding protein 280) (FLNA)
**cell cycle/cell death regulators**					
0.20	28.63	5.81	0.00	NM_015076.3	cyclin-dependent kinase (CDC2-like) 11 (CDK11)
0.22	36.33	7.84	0.01	NM_145341.2	programmed cell death 4 (neoplastic transformation inhibitor) (PDCD4)
0.22	27.59	6.12	0.01	NM_199246.1	cyclin G1 (CCNG1)
0.36	6.44	2.32	0.02	NM_015032.1	androgen-induced proliferation inhibitor (APRIN)
3.71	0.55	2.04	0.01	NM_199076.1	cyclin M2 (CNNM2)
4.11	1.18	4.84	0.05	NM_052988.2	cyclin-dependent kinase (CDC2-like) 10 (CDK10)
6.23	2.42	15.09	0.02	NM_005255.1	cyclin G associated kinase (GAK)
7.74	1.12	8.69	0.01	NM_001760.2	cyclin D3 (CCND3)
11.99	0.41	4.89	0.04	NM_001348.1	death-associated protein kinase 3 (DAPK3)
**defense/immunity**					
0.19	253.54	49.26	0.03	NM_002727.2	proteoglycan 1, secretory granule (PRG1)
0.25	49.26	12.44	0.00	NM_012329.1	monocyte to macrophage differentiation-associated (MMD)
0.44	311.28	136.65	0.03	NM_004048.2	beta-2-microglobulin (B2M)
2.35	4.10	9.61	0.04	NM_000600.1	interleukin 6 (interferon, beta 2) (IL6)
2.67	61.91	165.22	0.02	NM_002127.3	HLA-G histocompatibility antigen, class I, G (HLA-G)
4.17	43.87	183.09	0.01	NM_021034.1	interferon induced transmembrane protein 3 (1-8U) (IFITM3)
4.37	3.69	16.12	0.03	NM_006084.3	interferon-stimulated transcription factor 3, gamma 48kDa (ISGF3G)
4.78	47.43	226.89	0.04	NM_024298.2	leukocyte receptor cluster (LRC) member 4 (LENG4)
5.13	17.54	89.94	0.00	NM_003641.2	interferon induced transmembrane protein 1 (9-27) (IFITM1)
6.26	21.24	133.04	0.03	NM_005516.3	major histocompatibility complex, class I, E (HLA-E)
9.12	2.94	26.83	0.03	NM_005101.1	interferon, alpha-inducible protein (G1P2)
**unknown/other**					
0.26	17.20	4.41	0.05	NM_016299.1	heat shock 70kDa protein 14 (HSPA14)
0.28	7.91	2.25	0.01	NM_033285.2	tumor protein p53 inducible nuclear protein 1 (TP53INP1)
0.32	16.21	5.26	0.00	NM_015344.1	leptin receptor overlapping transcript-like 1 (LEPROTL1)
3.00	7.46	22.39	0.00	NM_005345.4	heat shock 70kDa protein 1A (HSPA1A)
4.37	90.74	396.54	0.02	NM_001540.2	heat shock 27kDa protein 1 (HSPB1)
22.26	0.17	3.79	0.01	NM_014567.2	breast cancer anti-estrogen resistance 1 (BCAR1)
